# Bitter Taste Stimuli Induce Differential Neural Codes in Mouse Brain

**DOI:** 10.1371/journal.pone.0041597

**Published:** 2012-07-23

**Authors:** David M. Wilson, John D. Boughter, Christian H. Lemon

**Affiliations:** 1 Department of Pharmacological and Physiological Science, Saint Louis University School of Medicine, Saint Louis, Missouri, United States of America; 2 Department of Anatomy and Neurobiology, University of Tennessee Health Science Center, Memphis, Tennessee, United States of America; German Institute for Human Nutrition, Germany

## Abstract

A growing literature suggests taste stimuli commonly classified as “bitter” induce heterogeneous neural and perceptual responses. Here, the central processing of bitter stimuli was studied in mice with genetically controlled bitter taste profiles. Using these mice removed genetic heterogeneity as a factor influencing gustatory neural codes for bitter stimuli. Electrophysiological activity (spikes) was recorded from single neurons in the nucleus tractus solitarius during oral delivery of taste solutions (26 total), including concentration series of the bitter tastants quinine, denatonium benzoate, cycloheximide, and sucrose octaacetate (SOA), presented to the whole mouth for 5 s. Seventy-nine neurons were sampled; in many cases multiple cells (2 to 5) were recorded from a mouse. Results showed bitter stimuli induced variable gustatory activity. For example, although some neurons responded robustly to quinine and cycloheximide, others displayed concentration-dependent activity (*p*<0.05) to quinine but not cycloheximide. Differential activity to bitter stimuli was observed across multiple neurons recorded from one animal in several mice. Across all cells, quinine and denatonium induced correlated spatial responses that differed (*p*<0.05) from those to cycloheximide and SOA. Modeling spatiotemporal neural ensemble activity revealed responses to quinine/denatonium and cycloheximide/SOA diverged during only an early, at least 1 s wide period of the taste response. Our findings highlight how temporal features of sensory processing contribute differences among bitter taste codes and build on data suggesting heterogeneity among “bitter” stimuli, data that challenge a strict monoguesia model for the bitter quality.

## Introduction

We commonly describe our taste experience using sensory categories, including sweet, salty, sour, and bitter. These categories, or qualities, have served diverse purposes in gustatory neurobiology research, from a convenience for stimulus classification to the basis for theories on the neural code for taste. One category could sufficiently describe the percept of a group of taste chemicals only if all of these stimuli induce a singular qualitative sensation and neural code [1]; i.e., they are monoguesic [2].

Bitter taste stimuli are structurally diverse chemicals sensed by a relatively large family of independent taste receptors, coined T2R [3]–[5]. T2R receptors can be selective for particular bitter stimuli or broadly responsive across diverse bitter ligands [6]–[10]. Some evidence suggests that all T2R receptors are expressed by one type of taste receptor cell in taste buds of the oral cavity [3], [5], predicting that all bitter stimuli should elicit a singular neural code. Although highly intercorrelated activity to select bitter stimuli has been suggested and revealed by behavioral [11] and neural [12], [13] studies, there is debate over whether all bitters induce a unitary neural signal and percept. Molecular studies of mouse and human taste papillae have revealed heterogeneous expression of T2R receptors across taste bud cells (TBCs) [4], [14]. Functional imaging studies show TBCs from outbred rats respond differentially to bitter stimuli such as cycloheximide, sucrose octaacetate (SOA), and quinine [15]. Neurophysiological recordings from outbred rodents show variability in peripheral [16], [17] and brain stem [18], [19] gustatory neural responses to bitters like quinine, denatonium, and cycloheximide. Moreover, psychophysical data from humans [20]–[22] and rodents [23], [24] show wide variation in sensitivity to diverse bitter stimuli and rats can discriminate between select bitter stimuli in taste detection paradigms [25], which would follow from differences in gustatory neural codes among bitters.

Taste electrophysiological data focused on central bitter coding have been obtained hitherto from genetically heterogeneous animals, in which inter-individual differences likely contribute variance to bitter sensitivity [20], [22], [23]. Here, we recorded taste-evoked activity to a diverse panel of bitters from neurons in the nucleus tractus solitarius (NTS) in two lines of mice with genetically fixed bitter taste profiles. Mice included an isogenic inbred strain and a congenic bitter “taster” line, each possessing a distinct bitter sensitivity profile. In both lines, avoided bitter stimuli induced differential neural codes due to divergence of responses during an early period of taste stimulation. This effect was found for natural bitter stimuli of different toxicity, suggesting potential ecological significance to divergent bitter codes. Our findings further question the singularity of neural representations for “bitter” taste stimuli and highlight how temporal features of neural activity contribute variations in chemosensory responses [26]–[28].

## Methods

### Ethics statement

All procedures were performed on mice under anesthesia in accordance with National Institutes of Health guidelines and protocols reviewed and approved by the Institutional Animal Care and Use Committee of St. Louis University (Permit Number: 2014).

### Mouse lines

All mice were housed in a vivarium that maintained a 12 h light/dark cycle and an ambient temperature of ∼23°C. Food and water were available ad libitum. Two mouse lines were tested to assess repeatability of observed effects across strains. One of the lines used was the inbred C3HeB/FeJ (C3) strain (8 males, 15 females; mean body weight, in g = 38.3±1.5 s.e.m.). C3 mice show behavioral avoidance towards bitter stimuli such as quinine and denatonium benzoate [29], [30] and are particularly sensitive to the bitter tastant cycloheximide [6], [31]. C3 mice used to establish a local inbred colony were purchased from The Jackson Laboratory (Bar Harbor, ME).

The second mouse line used was the congenic C3.SW-*Soa* ^a^ (C3.SW) strain (15 males, 7 females; mean body weight  = 39.7±1.1). These mice have the genomic background of the C3 line but possess an introgressed segment of distal Chr 6 from SWR/J mice harboring the “taster” allele of the genetic locus *Soa*, which confers sensitivity to the bitter acetylated sugar SOA [32], [33]. This locus is linked to a cluster of genes encoding T2R taste receptors for bitter stimuli [3], [4], [33]. C3.SW mice strongly avoid SOA, whereas C3 mice are relatively insensitive to this stimulus [29], [30], [32], [34]. Yet, as with the C3 line, C3.SW mice detect and avoid quinine, denatonium benzoate, and cycloheximide, albeit with some differences between lines in the strengths of these aversions [29], [30]. C3.SW taster mice were transferred from the University of Tennessee Health Science Center and a local colony was established and maintained through inbreeding. C3.SW mice selected randomly from our colony for behavioral phenotyping using 48-hr two bottle intake tests with 0.1 mM SOA and water [29], [32] all showed criterion avoidance of SOA associated with the SWR/J *Soa* ^a^ taster allele (criterion: an SOA preference ratio of <0.15, as given by the amount of SOA consumed over the total amount of SOA and water consumed) [32]; random C3 mice were indifferent to 0.1 mM SOA in these tests, as expected.

### Single-neuron electrophysiology

Selection of C3 and C3.SW mice for daily recordings was interleaved when possible. For each mouse, anesthesia was induced using a combination of urethane (1.2 g/kg, i.p.) and pentobarbital (40 mg/kg, i.p.). Anesthesia promoted recording of sensory neural responses in the absence of non-specific influences, such as differences in behavioral state across animals [35]. A tracheal cannula was inserted to facilitate ease of breathing during oral solution flow. The lower incisor was trimmed using rongeurs. Mice were positioned in a non-traumatic head holder that angled the snout ∼25° downward. A silk thread was run caudal of the lower incisor, pulled tight to deflect the mandible downward, and then fixed in place to keep the mouth open. The tongue was protruded from the mouth by a small ventral suture. Body temperature was kept at ∼37°C by a heating pad. A portion of the occipital bone was removed and parts of the cerebellum were gently aspirated to allow vertical access to the medulla. The rostral, taste-sensitive region of the NTS was targeted using vascular and anatomical landmarks on the dorsal surface of the brain stem [36].

Trains of extracellular action potentials were recorded from taste-sensitive NTS neurons using conventional electrophysiological methods. Tungsten microelectrodes (*z* = 2 to 5 MΩ, FHC, Bowdoinham, ME) sampled unit electrophysiological activity that was band-passed filtered (0.3 to 10 kHz), AC amplified (Grass P511, high-*z* probe), and monitored on an oscilloscope and loudspeaker. A hydraulic micromanipulator advanced the electrode ventrally through brain tissue. Neural activity was digitally sampled (at 25 kHz, 1401 interface and Spike2 software, CED, Cambridge, UK) and spikes generated by individual neurons were identified by experimenter and software based on waveform consistency. Digital records were saved for offline analysis.

At the end of data recording, a weak electrical current (100 μA/2 to 3 s) was passed through the recording electrode to create an electrolytic lesion at the last position of the electrode's tip. For mice where multiple neurons were sampled, only one lesion was made at the location of the last cell acquired. Anesthetized mice were then overdosed with sodium pentobarbital (130 mg/kg, i.p.) and perfused transcardially with isotonic saline followed by a mixture of 4% paraformaldehyde and 3% sucrose. Brains were removed and stored at least overnight in a mixture of 4% paraformaldehyde and 20% sucrose. Brain stems were cut by microtome into coronal sections (40 μm) mounted onto slides and stained with thionin. Lesions were compared against an atlas of the mouse brain [37] to determine electrode placement.

### Taste stimuli

Twenty-six stimuli were tested. Bitter stimuli included concentration series of quinine, denatonium, cycloheximide, and SOA (Table 1). Testing multiple concentrations determined how response phenomena were influenced by stimulus intensity and facilitated assessment of repeatability of bitter-induced responses over multiple trials. Acquiring multiple tastant responses was critical for analyses of time-dependencies in neural activity, as carried out below. Also tested were propylthiouracil, sugar and sweet-like stimuli (sucrose, saccharin, and ethanol), Na^+^ salts (NaCl, NaNO_3_), acids (HCl, citric acid) and purified water. Stimuli (Sigma, St. Louis, MO) were dissolved in purified water and tested at room temperature. Once isolated, neurons were first stimulated with oral delivery of stimuli representative of different tastes including sucrose, NaCl, HCl (see Table 1 for concentrations), 10 mM quinine, and water, presented in random order. Concentration series of the bitter stimuli quinine, denatonium, cycloheximide, and SOA and a single concentration of propylthiouracil were tested next. The ordering of bitter stimuli was randomized, but concentration series for bitter tastants were tested in ascending order. Following the bitter stimuli, saccharin, ethanol, NaNO_3_, and citric acid were presented in randomized order. For some neurons, the prototype stimuli were retested following completion of all trials to ensure stability of recording.

**Table 1:** Taste stimuli, concentrations, and abbreviations.

Stimulus	Concentration(s)	Abbreviation for figures
propylthiouracil	1 Mm	pr
quinine	0.3, 1, 3, and 10 mM	qui
denatonium benzoate	0.1, 0.3, 1, 3, and 10 mM	den
cycloheximide	0.3, 3, 30, 100, and 300 μM	cyx
sucrose octaacetate	0.1, 0.3, and 1 mM	soa
water	n/a	w
sucrose	500 mM	su
saccharin (Na^+^)	5 mM	sa
ethanol	40%	e
NaCl	100 mM	na
NaNO_3_	100 mM	nn
HCl	10 mM	h
citric acid	10 mM	ci

**Figure 1:**
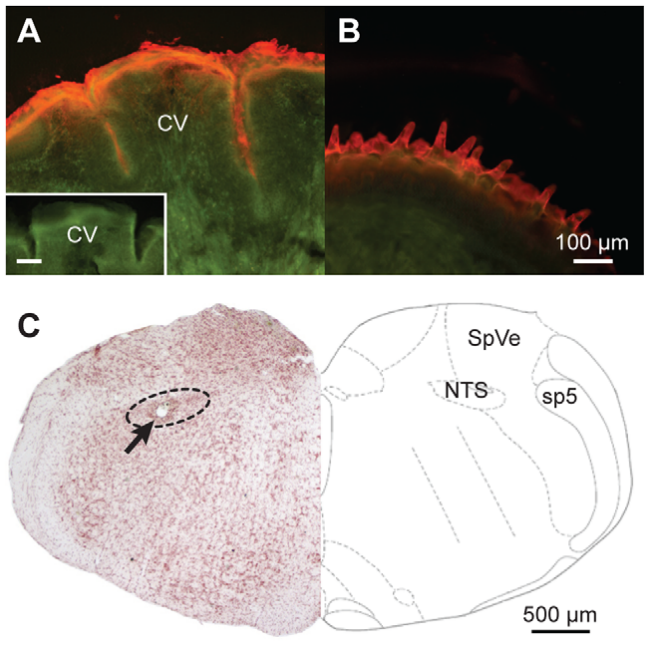
Photomicrographs illustrating assessment of oral stimulation field and histological analysis of recording electrode location. The top panel shows images of coronal sections (40 μm) through (**A**) the posterior circumvallate (CV) region and (**B**) anterior portion of two mouse tongues. Tongues were immediately removed and sectioned following oral delivery of fluorescent dye through our taste presentation system. Fluorescent dye, red under our filter settings, covered the anterior and posterior tongue surface and invaded the posterior tongue CV crypts housing taste receptors. Inset in A is a cross-section through the CV region of a tongue that was not stimulated with dye and shows the tongue does not naturally fluoresce. Inset scale bar is 100 μm. Photomicrographs were adjusted in Adobe Photoshop CS4 software (version 11.0.2; Adobe Systems, Inc., San Jose, CA) using levels, brightness, and contrast. (**C**) Left, image of a coronal section (40 μm) through mouse brain stem showing an electrolytic lesion made at a recording location (arrow). Schematic on the right (adapted from [37]; with publisher's permission) shows the location of the NTS relative to select landmarks, including the spinal vestibular nucleus (SpVe) and spinal trigeminal tract (sp5).

Stimuli were stored in airtight glass bottles (dark glass bottles were used for light-sensitive compounds) and were delivered one at a time to the mouse oral cavity using a funnel/gravity flow system, the basics of which have been described previously [36]. For most mice, the oral field stimulated by taste chemicals delivered through the gravity flow system was deduced at the end of recording by flowing dye (thionin or Evans blue) in the same manner that taste solutions were presented. Oral staining was inspected under a microscope after fixative perfusion of the animal and subsequent dissection of the oral cavity. Dye was found consistently on the anterior and posterior tongue, nasoincisor duct, and soft palate. For some mice stimulated with the fluorescent dye Evans blue, the tongue was removed following perfusion, immediately cut by microtome into coronal sections (40 to 80 μm), and the posterior tongue circumvallate folds housing taste receptors were inspected with a fluorescence microscope. Evans blue clearly invaded the circumvallate folds (Figure 1A), suggesting taste solutions reached circumvallate taste receptors; this staining was comparable to that observed on the anterior tongue surface (Figure 1B). Moreover, we routinely observed strong neural responses to the bitter tastant cycloheximide, which in rats is ineffective for taste receptors on the anterior tongue [13] but a strong stimulant for NTS neurons receiving input from posterior oral fields [18], [19]. Thus, our experiments aimed to stimulate the entire mouth with taste solutions. This “whole mouth” technique was facilitated by the small size of the mouse oral cavity.

Stimulus trials were 15 s in duration, but divided into 5 s epochs. A trial began with a room temperature purified water rinse delivered from trial onset to 5 s into the trial. This served to pre-adapt and control for any mechanical component to oral stimulation. Solution flow then was switched to the stimulus, which was delivered for 5 s. Flow was switched back to purified water for the final 5 s. An inline electronic 3-way fluid valve controlled by the data acquisition system accomplished precise switching of stimulus flow. In between trials, all solution delivery tubing and the fluid ports of the valve were flushed with at least 125 ml of purified water. This rinse also bathed the mouse oral cavity to ensure removal of the stimulus on the preceding trial, precluding adaptation effects. The inter-trial interval was approximately 90 to 120 s and was sufficient to allow cells to return to baseline activity levels. Mice did not ingest solutions, which fell into a drain positioned beneath the mandible.

On each trial, there was a lag from the computer signal that turned on taste stimulus flow until oral delivery of the stimulus due to the time it took solutions to move through the passageways of the fluid valve and oral delivery tubing. To estimate this lag, we ran a set of mock trials (without a mouse) where warmed water (∼30°C) was presented through the delivery system in the same manner as a taste stimulus (i.e., preceded by a room temperature, ∼22°C, rinse) and the time lag until solution outflow temperature increased was captured. A thermocouple sensor placed at the end of the oral delivery tube monitored near-instantaneous changes in outflow temperature; the thermocouple circuit was linked to our data acquisition system to measure change in temperature against time. The lag from the stimulus “on” signal to a temperature increase estimated how long it took solutions to flow through the valve and oral delivery tubing. This lag was 369±8 ms (mean ± s.e.m.), over 20 trials. There was additional lag in neuronal responses relative to stimulus onset due to the time required for stimuli to engage taste receptors, peripheral signal integration, and neuronal conduction velocity to the brain stem.

### Data analysis

Gustatory activity by single NTS neurons was analyzed within each mouse line in two phases. Phase I focused on spatial characteristics of taste activity quantified by “net response”, operationally defined as the number of spikes evoked during the 5 s taste stimulation period minus the number of spikes that arose during the 5 s pre-stimulus (baseline) period. Net responses to a stimulus of fixed concentration measured repeatedly from one neuron were averaged. Net response data were analyzed by ANOVA (SPSS v 17.0, IBM, Somers, NY), where applicable; α was set to 0.05. Sex was not a factor in analyses, as sex effects on bitter taste sensitivity have not been reported in studies of C3 and C3.SW mice [29], [30], [34]. Net response data also were analyzed through correlational methods involving Pearson's product-moment correlation coefficient (*r*) and multivariate techniques, including hierarchical clustering and metric multidimensional scaling (MDS).

Hierarchical clustering was used in two ways. First, this technique was applied to define traditional neuron groups in each mouse line for plotting how bitter activity was, on average, distributed amongst them. Cluster analyses here were performed on matrices of correlation distances (1–*r*) among neurons computed from their net responses to stimuli representative of different taste categories: (in mM) 500 sucrose, 100 NaCl, 10 HCl, 10 quinine, and 0.03 cycloheximide. Correlation distance captures relationships in neural tuning (e.g., similarities in the “shapes” of neural tuning profiles) and can be insensitive to response level. The unweighted average distance amalgamation schedule was used. The number of resulting clusters was given by a “scree” plot of cluster distances against amalgamation steps and assessing at which step the plot “elbowed” [19], [36].

Secondly, hierarchical clustering was applied to sort neurons within each mouse line by their net responses to all bitter stimuli. These sorts were then used in conjunction with heatmap plots to visualize bitter response data across all neurons. Here, cluster analyses used Ward's amalgamation schedule and Euclidean distances between cells, as computed from their bitter activity profiles. Euclidean distance is appropriate to sort neurons by bitter response magnitude, as this distance is sensitive to response level.

MDS produced plots that captured dissimilarity between net responses to taste stimuli across neurons within each line. To avoid local minima, MDS was repeated 50 times using random starting configurations and the solution showing the overall least stress (i.e., badness-of-fit to the data) was used for interpretation. Similar solutions and stress values were achieved on the majority of the replicate runs.

Phase II of data analysis focused on spatiotemporal properties of neural population activity to bitter stimuli. Here, analyses were performed on neural spike data “time-sliced” into consecutive brief windows of activity arising during the course of sensory stimulation [26], [28], [38]–[40]. To do this, taste-induced spike trains by single neurons were translated into vectors of 500 ms bins, where each bin held the number of spikes occurring during a half-second epoch of a stimulus trial. Principal components (PC) and correlation analyses were applied to across-neuron response vectors for stimuli, all aligned by trial onset, to assess differences and similarities in bitter taste responses over consecutive half-second periods of taste stimulation. Other bin sizes were also tried (e.g., 200 ms, 250 ms, 1 s) and results were similar to those reported herein, albeit neural response vectors binned to 100 ms or less became too sparse for effective analysis.

For phase II, PC analysis was used to generate visualizable “maps” of spatiotemporal codes to different stimuli. Visualizations similar to those had under PC analysis were also achieved under other dimensionality-reduction methods, such as metric MDS. However, MDS analysis of response time course data based on correlation distance (1–*r*) showed inconsistencies for mapping sequential, low-level patterns of activity. This anomaly was attributable to the high sensitivity of the Pearson correlation to variability in low-level activity patterns and the distortions that come along with using correlation distance to track such responses [41]. This distortion was not observed under PC analysis, which is commonly used to visually represent spatiotemporal neural codes in chemosensory systems [26], [39], [40].

Multivariate procedures in phases I and II were carried out using custom code in MATLAB (release 2011a, The MathWorks, Natick, MA). Routines from the Statistics and Bioinformatics Toolboxes for this platform were used.

## Results

**Figure 2:**
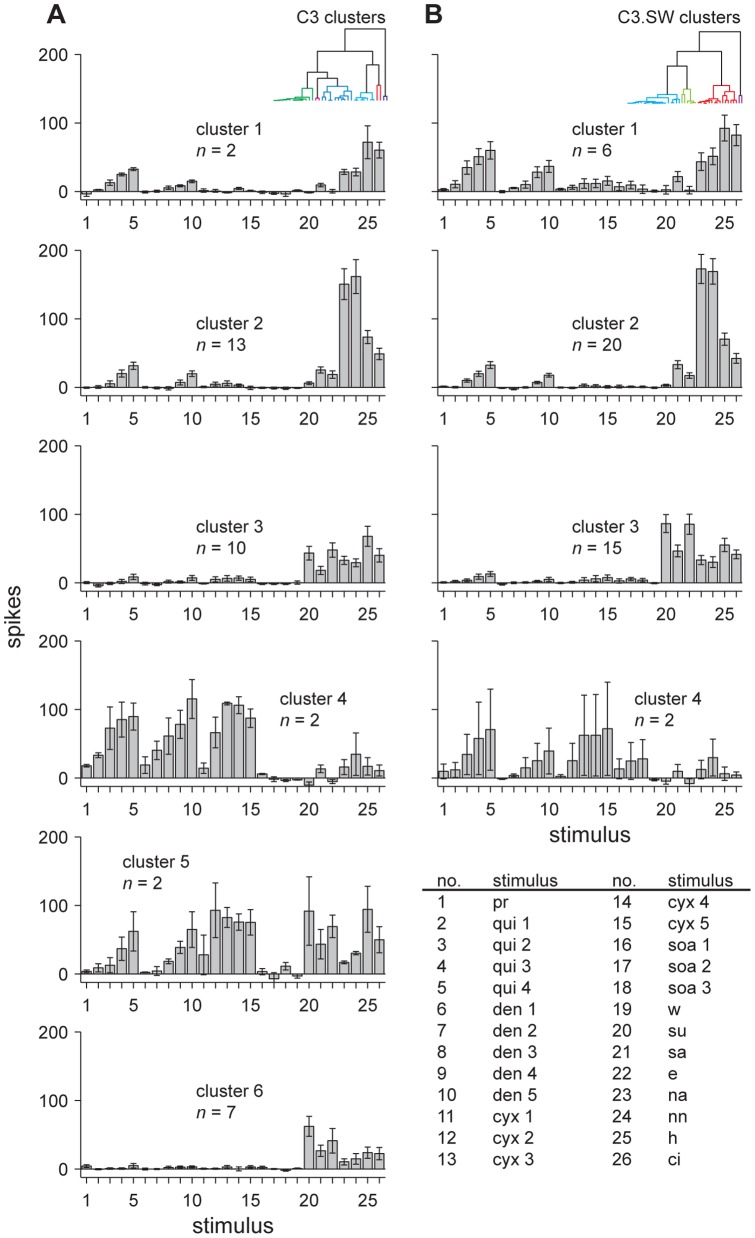
Definition of neural clusters. Groupings of NTS neurons in C3 (**A**) and C3.SW (**B**) mice defined by hierarchical clustering of activity to stimuli representative of different taste categories. Y-bars represent mean ± s.e.m. responses (net spikes in 5 s). Dendrograms showing cluster recovery in each line are depicted by insets near the top of each panel. Numbers along the abscissae denote stimuli (legend). In the legend, numbers in stimulus abbreviations indicate concentrations from lowest (e.g., 1) to highest (e.g., 5), where applicable (Table 1).

Trains of action potentials were recorded from 36 NTS neurons in C3 mice and 43 NTS cells in C3.SW mice. All 79 neurons were tested with all 26 stimuli in Table 1 at least once. A total of 1049 stimulus trials were acquired from C3 neurons; 1236 trials were recorded from C3.SW cells. Baseline, pre-stimulus firing rates were low for units in both mouse lines (in spikes s^−1^ ± s.e.m: C3, 1.1±0.04; C3.SW, 1.5±0.1). Neurons remained active and stable throughout data collection trials. Net responses to representative stimuli ([in mM] 500 sucrose, 100 NaCl, 10 HCl, and 10 quinine) were the same from initial measurement to retesting these tastants following completion of trials for all stimuli in Table 1 (data from 27 neurons, repeated-measures ANOVA, n.s. time × stimulus interaction, *F*_3,78_ = 2.4, *p* = 0.08). Across all cells, the largest net taste response observed was 334 spikes. The lowest net response was −22 spikes, albeit inhibitory (i.e., below baseline) responses such as this were rare. Only 1% of all net responses were lower than −10 spikes. Thus, excitation dominated taste activity, as observed in many other neurophysiological studies of gustatory NTS. Electrode positioning indeed targeted the NTS (Figure 1C). Although not all tissue was recovered, 28 recording sites were reconstructed in this nucleus. Figure 2 shows the distribution of bitter sensitivity across C3 and C3.SW neuron groups recovered by hierarchical cluster analysis.

### Phase I: Characterizing spatial responses to bitter stimuli by mouse NTS neurons

**Figure 3:**
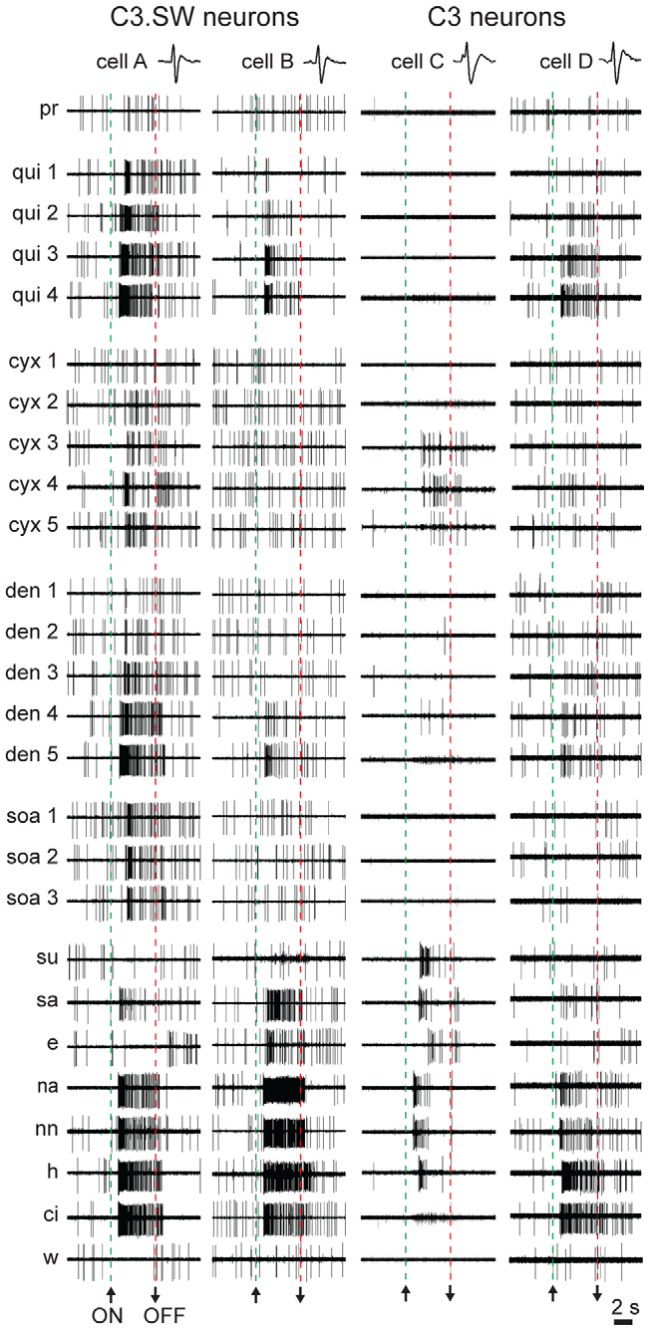
Raw response data from taste-sensitive neurons. Digital oscilloscope sweeps showing electrophysiological activity to all stimuli recorded from two C3.SW cells (A and B) and two C3 neurons (C and D). The C3 neurons were recorded in series from one mouse; C3.SW cells are from different mice. The stimulus tested during each sweep is abbreviated (Table 1) along the left margin. Where applicable, numbers in stimulus abbreviations indicate concentrations from lowest (e.g., 1) to highest (e.g., 5), as in Table 1. Upward and downward arrows at the bottom of each sweep stack indicate stimulus onset and offset, respectively.

**Figure 4:**
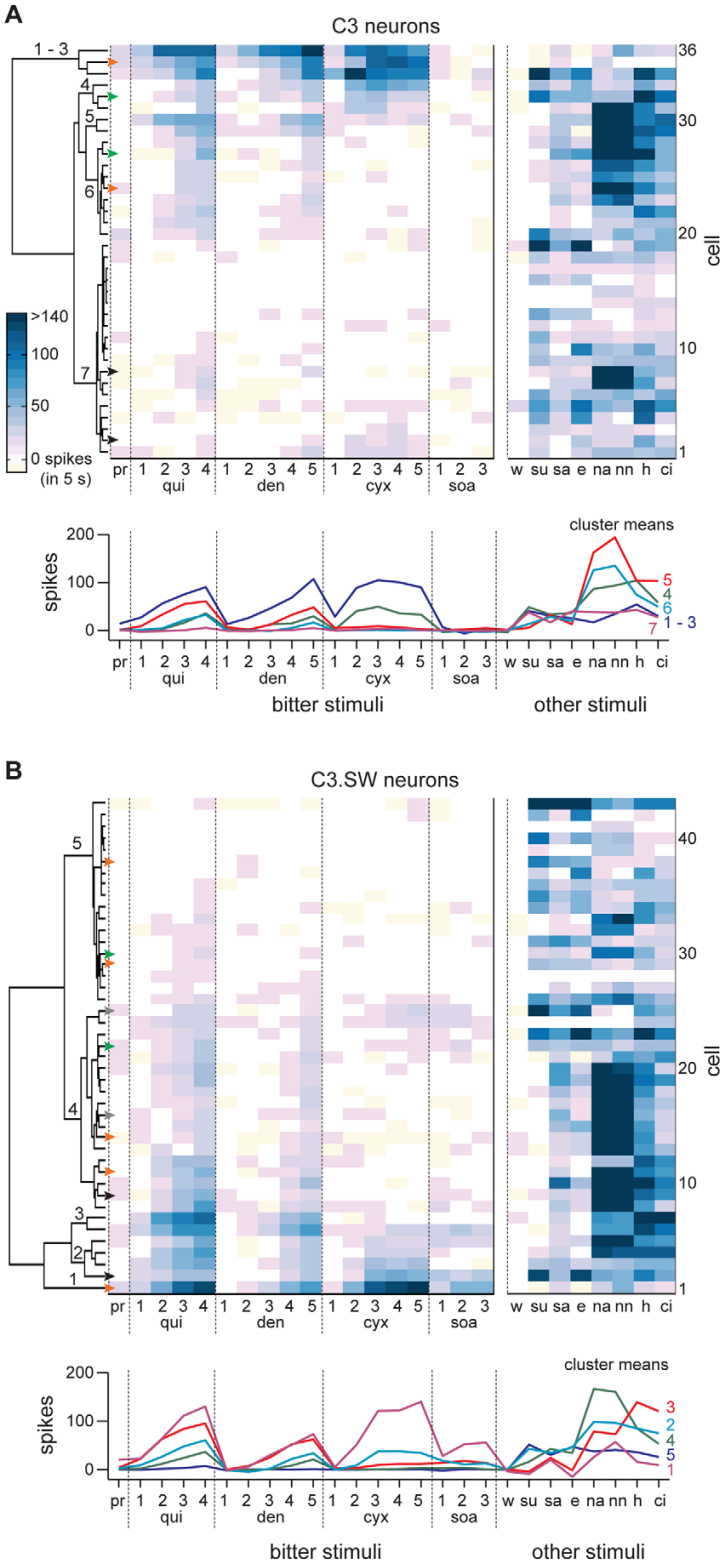
Neural responses to bitter and other stimuli. Heatmaps showing the net 5 s response to each of 26 taste stimuli (abscissae) across all 36 C3 (**A**) and 43 C3.SW (**B**) neurons (ordinates). The heat scale in panel **A** gives response spike density for panels **A** and **B**. Neurons are sorted within mouse line by cluster analysis of activity to all concentrations of quinine, denatonium, cycloheximide, sucrose octaacetate, and propylthiouracil. Pairs of arrowheads of the same color along the base of each dendrogram highlight neurons that were recorded from one mouse and showed differential sensitivity to bitter stimuli (e.g., cells marked by green arrowheads were recorded from one mouse; pair in black from another, etc.). Orange arrowheads along the base of the dendrogram in panel **B** denote response data from five neurons recorded in series from one C3.SW mouse. Numbers on dendrograms mark neural clusters determined by “scree” plots. Table 1 gives stimulus abbreviations. Numbers above abbreviations for bitter stimuli indicate concentrations from lowest (e.g., 1) to highest (e.g., 5), as in Table 1. Plots of average activity in each cluster are given below dendrograms; numbers color-matched to each plot indicate cluster(s).

Data from individual units in Figure 3 illustrate an effect common to our sampled neuronal populations: bitter-sensitive cells in both mouse lines possessed variable tuning profiles to bitter tastants. For example, the C3.SW neuron labeled A in this figure showed robust responses across concentrations of quinine, cycloheximide, denatonium, and SOA, whereas C3.SW cell B showed clear activation to only quinine and denatonium. These neurons were sampled from different mice, albeit differential tuning to bitter stimuli also arose across multiple cells recorded from one mouse. Neurons C and D in Figure 3 were recorded sequentially from a C3 mouse. Unit C of this pair responded consistently to only cycloheximide among bitters, whereas cell D appeared relatively insensitive to this input, as compared to baseline, but was activated by concentrations of quinine and denatonium. The position of the taste delivery device in the mouth was not adjusted in between sampling these cells or other neurons recorded in series from one animal. Other examples of differential tuning to bitter stimuli across multiple neurons recorded from one mouse are shown in Figure 4 (colored arrowheads, see legend). That select bitter stimuli activated some but not other bitter-responsive neurons in one mouse afforded within-animal control that insensitivity to these inputs was not attributable to ineffective stimulus concentrations or ineffective stimulation of taste receptors.

### Bitter stimuli induce variable patterns of spatial activity across C3 and C3.SW neurons

Clustergrams used to sort neurons within line by their net responses to all concentrations of quinine, denatonium, cycloheximide, SOA, and 1 mM propylthiouracil are plotted in Figure 4. In this figure, heatmaps portray the activity of all neurons to all stimuli and concentrations tested. In both mouse lines, subsets of bitter-sensitive neurons responded very differently to bitter stimuli. Among the 7 neuronal groupings that emerged from hierarchical clustering of C3 neurons, the majority of cells in clusters 1 through 5 in Figure 4A clearly showed strong responses across concentrations of quinine, denatonium, and cycloheximide. The low *n* of each of these clusters precluded statistical analysis of bitter activity within groups, albeit the general responsiveness of these neurons to concentrations of quinine, denatonium, and cycloheximide was clearly observable (Figure 4A). On the other hand, C3 cells in cluster 6 (Figure 4A) showed concentration-dependent responses to quinine (effect of concentration, *F*_3,24_ = 36.5, *p*<10^−3^) and denatonium (effect of concentration, *F*_4,32_ = 17.7, *p*<10^−3^) but not to cycloheximide (n.s. effect of concentration, *F*_4,32_ = 0.3, *p* = 0.9), an ineffective stimulant for the majority of units in this cluster.

**Figure 5:**
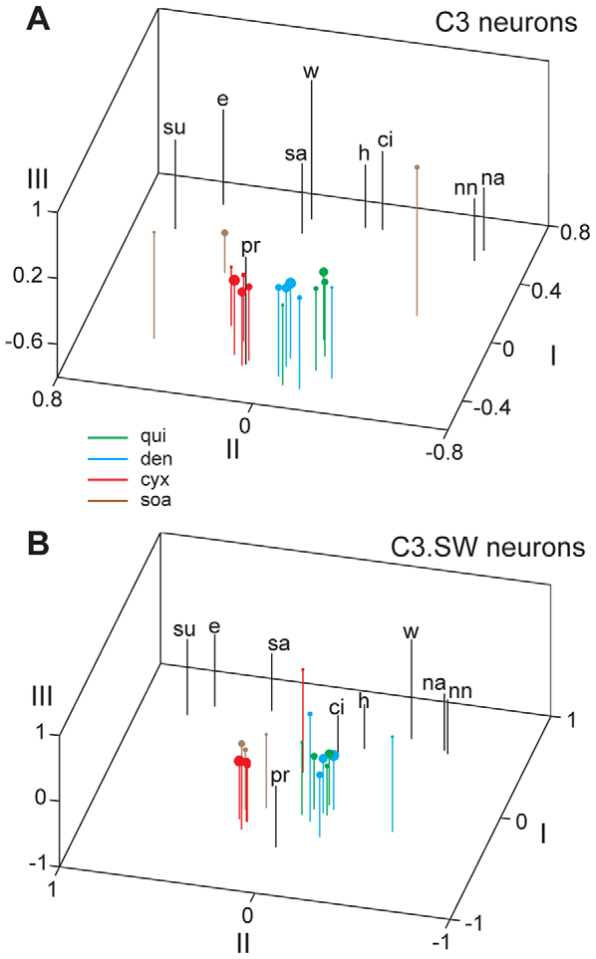
Clustering of taste responses to bitter and other stimuli. Three-dimensional plots showing the outcome of multidimensional scaling of net responses to all taste stimuli across 36 C3 (**A**) and 43 C3.SW (**B**) neurons. Table 1 gives stimulus abbreviations used in each space. Responses to cycloheximide, quinine, denatonium, and SOA are color-coded (legend in panel **A**), and responses to increasing concentrations of these stimuli (Table 1) are respectively represented by points/circles of increasing diameter. Dimensions of plots represent arbitrary units.

Patterns of activity to bitter stimuli by C3 neurons were further assessed using MDS analysis. MDS was applied to distance (1–*r*) matrices that quantified dissimilarity (and, concomitantly, similarity) between taste responses across neurons within mouse line. MDS reduced the high dimensionality of these matrices into lower dimensional, visualizable representations that captured relationships between patterns of activity evoked by taste stimuli. Several noteworthy observations arose from MDS applied to data from C3 cells. First, responses to concentrations of quinine and denatonium tended to cluster in scaling space (Figure 5A), reflecting a common pattern of activity to these inputs. Computation of pairwise Pearson correlations between responses to all concentrations of these stimuli showed that 3 mM quinine and 10 mM denatonium induced the most correlated (*r* = +0.94) response patterns. The strength of this correlation is highlighted by its coefficient of determination (*r* ^2^ = 0.88), which revealed that a simple linear function accounted for a large majority, 88%, of the variance in responses to these stimuli.

Considering cycloheximide, multiple concentrations of this input induced responses in C3 neurons that were clustered in MDS space but separated from activity to quinine and denatonium (Figure 5A). The highest correlation for C3 activity to cycloheximide (30 μM) and denatonium (10 mM) was +0.87, and the strongest correlation between C3 responses to cycloheximide (0.1 mM) and quinine (0.3 mM) was +0.75; both of these *r* values were significantly lower than the strongest correlation between C3 responses to quinine and denatonium, +0.94, reported above (tests of *r*  =  ρ, |*z*_obt_| >2.3, *p*<0.05). Further, squaring these correlations showed that a linear function accounted for a maximum of 76% of the variance in C3 neural activity to cycloheximide and denatonium (*r* ^2^ = 0.76) and only 56%, at best, of the response variance to cycloheximide and quinine (*r* ^2^ = 0.56), substantially lower than the highest association between responses to quinine and denatonium (*r* ^2^ = 0.88). In summary, the “bitter” stimuli cycloheximide, quinine, and denatonium induced responses in C3 cells of variable similarity. C3 mice are relatively insensitive to SOA [29], [30], [32], [34], which induced weak activity across C3 cells (Figure 4A). The scattering of SOA responses in the C3 scaling space may reflect correlative “noise” and not a neural coding effect.

Variable activity to bitter stimuli was also found for neurons in C3.SW mice. Hierarchical clustering produced 5 neuronal clusters in this line. C3.SW cells in clusters 1 through 3 in Figure 4B generally showed robust responses to quinine, denatonium, and cycloheximide, across concentrations, and the majority of these cells also showed sensitivity to SOA, which is detectable by C3.SW mice in orosensory tests [30], [34]. On the other hand, bitter-sensitive cells in C3.SW cluster 4 (Figure 4B) showed concentration-dependent responses to quinine (effect of concentration, *F*_3,51_ = 50.1, *p*<10^−3^) and denatonium (effect of concentration, *F*_4,68_ = 53.6, *p*<10^−3^), but not to cycloheximide (n.s. effect of concentration, *F*_4,68_ = 0.8, *p* = 0.5) or SOA (n.s. effect of concentration, *F*_2,34_ = 1.3, *p* = 0.3). Cycloheximide and SOA were ineffective stimuli for several units in cluster 4, albeit all cells in this class showed measurable responses to quinine and denatonium. Similarly, several cells in C3.SW cluster 5 showed responses to quinine but not to cycloheximide or SOA (Figure 4B).

MDS also revealed heterogeneity among response patterns to bitters across C3.SW cells. Responses to salient concentrations of cycloheximide and SOA formed a tight cluster in scaling space largely separated from activity to quinine and denatonium (Figure 5B). C3.SW cells gave responses to quinine and denatonium that showed strong positive correlation, where activity to 3 mM quinine and 3 mM denatonium produced the highest correlation, +0.91 (*r* ^2^ = 0.83), across concentrations. However, lesser correlations were found when comparing responses to quinine and denatonium with activity to cycloheximide and SOA. For C3.SW cells, the strongest correlation noted for activity to cycloheximide (30 μM) and quinine (3 mM) was +0.70 (*r* ^2^ = 0.49), the strongest for cycloheximide (0.1 mM) against denatonium (3 mM) was +0.61 (*r* ^2^ = 0.38), the highest for SOA (1 mM) and quinine (0.3 mM) was +0.66 (*r* ^2^ = 0.43), and the strongest for SOA (0.3 mM) and denatonium (10 mM) was +0.63 (*r* ^2^ = 0.40). Moreover, all of these *r*-values were significantly lower than the maximal correlation, +0.91, noted for C3.SW activity to quinine and denatonium (tests of *r*  =  ρ, |*z*_obt_| >4.2, *p*<0.01). As found for C3 cells, “bitter” stimuli induced spatial response patterns of variable similarity in C3.SW neurons.

### Correlations involving responses to non-bitter stimuli

In each mouse line, there was high correlation between net responses to the Na^+^ salts NaCl and NaNO_3_ (C3, *r* = +0.98; C3.SW, *r* = +0.96). The Pearson correlation among activity to the acidic stimuli HCl and citric acid was +0.85 for C3.SW cells and +0.77 for C3 neurons. Although this C3 correlation appears reduced, it is important to note that, unlike bitters, correlations among responses to non-bitter stimuli pertained to only single concentrations of these inputs. Future tests using several concentrations of acids might reveal a higher correlation in the C3 line. Indeed, Pearson correlations ranging from +0.73 to +0.87 were found comparing net responses to multiple concentrations of HCl against 0.01 M citric acid recorded across 25 NTS neurons in C57BL/6J mice (data from [36]). Expectedly, the sweet and sweet-like stimuli sucrose, saccharin, and ethanol induced variably correlated responses in C3 (+0.52< *r* <+0.75) and C3.SW (+0.57< *r* <+0.89) neurons, as saccharin and ethanol induce cross-quality and – modal features. Unlike sucrose, saccharin engages sweet and bitter taste receptors [36], [42] and has both sweet and bitter tastes [43]. Ethanol is a stimulant of sweet taste pathways [44] and also somatosensory trigeminal afferents [45], which synapse onto NTS cells associated with taste and oral sensory processing [46]. Finally, correlations between non-bitter stimuli were of varied range (C3, −0.38< *r* <+0.61; C3.SW, −0.41< *r* <+0.48), as were correlations between all bitter and non-bitter inputs (C3, −0.31< *r* <+0.40; C3.SW, −0.42< *r* <+0.54).

### Phase II: Characterizing time-evolved responses to bitter stimuli by mouse NTS neurons

Results hitherto showed that taste stimuli usually assigned to a unitary “bitter” class induced varying spatial responses across central taste-sensitive neurons and replicated this finding across two mouse lines with unique bitter taste profiles. These analyses indexed spatial characteristics of neural activity over a long time window (5 s), which overlooked the contributions of early (phasic) and later periods of the taste response to bitter coding phenomena. To explore bitter coding in higher temporal detail, we characterized the time course of bitter responding by C3 and C3.SW cells using techniques drawn from the study of spatiotemporal coding by hypothetical neural ensembles in olfaction [26], [28], [38]–[40]. For our cells, spike trains were binned into rate envelope vectors, where contiguous bins of a vector held spike counts arising during contiguous 500 ms epochs of a taste trial. For each stimulus, response vectors across neurons were aligned in time by trial onset to form a series of sequential, half-second wide across-neuron response patterns that gauged how activity to that stimulus evolved over the course of taste stimulation.

### Bitter stimuli induce differential spatiotemporal responses across C3 and C3.SW neurons

**Figure 6:**
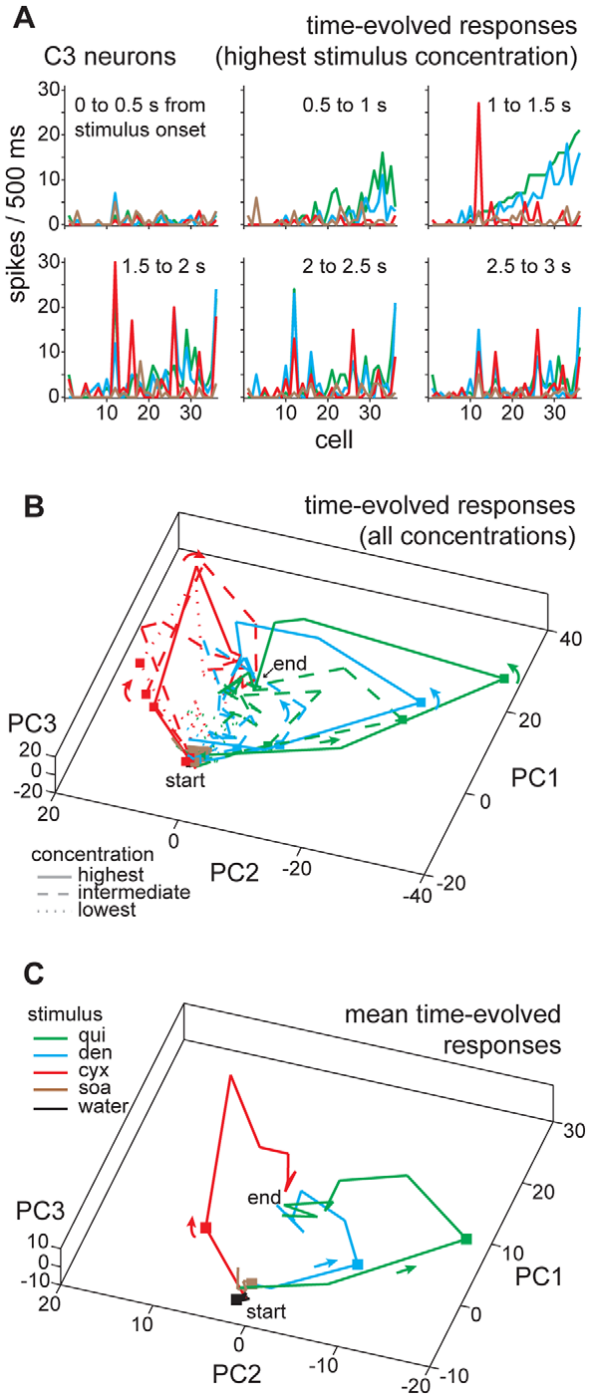
Modeling time dependencies in bitter coding by C3 neurons. (**A**) Plots showing sequential, 500 ms wide windows of taste activity (spike density per half-second, ordinates) across 36 C3 cells (abscissae) to the highest concentrations of quinine, denatonium, cycloheximide, and sucrose octaacetate. The time window of taste activity captured by each plot is indicated. Legend in **C** gives the stimulus associated with each colored response for all panels in this figure. (**B**) Three-dimensional plot showing the outcome of principal components (PC) analysis applied to sequential, 500 ms wide windows of activity (cf. panel **A**) across 36 C3 neurons during taste stimulation with all concentrations of quinine, denatonium, cycloheximide, sucrose octaacetate, and also water. Response windows from stimulus onset to offset (i.e., 0 to 5 s post stimulus) are represented. For each stimulus, PC-mapped points for sequential response windows are connected using color-coded lines, forming “paths” in the space describing time-evolved neural activity to bitter inputs. “Elbows” along each path represent points for response windows. Arrowheads indicate flow and sequencing of contiguous windows. Along each path, the point representing time-sliced activity arising 1 to 1.5 s post stimulus onset is marked by a square. Paths for activity to all low, intermediate and high concentrations (legend, concentrations as in Table 1) of each stimulus are shown; responses to intermediate concentrations are not differentiated. PC1, PC2, and PC3 explain 78% of the total response variance. The general locales of the “start” and “end” points for the trajectories are indicated. (**C**) Same as panel **B**, except that activity within each 500 ms response window was averaged over concentrations for each stimulus prior to PC analysis, highlighting global trends in the data. PC1, PC2, and PC3 explain 86% of the total response variance.

Figure 6A shows the evolution in half-second steps of across-neuron patterns of activity by C3 neurons during oral stimulation with the highest concentrations of quinine, denatonium, cycloheximide, and SOA; SOA, expectedly, induced only weak activity in cells recorded from C3 mice. In these plots, neurons are rank-ordered by their response to quinine in the 500 ms window residing 1 to 1.5 s from stimulus onset. During this period, quinine and denatonium evoked robust and similar patterns of activity that were clearly different from the pattern of response to cycloheximide, which induced relatively poor or null activity in many neurons with high sensitivity to quinine and denatonium (Figure 6A). Cycloheximide became a more effective stimulant for several neurons in the next 500 ms period of the response (1.5 to 2 s) and persisted to stimulate these units into later time windows. Several units giving sustained responses to cycloheximide also showed sustained activity to quinine and denatonium. Further, many units that showed early (from 0.5 to 1.5 s) activity to quinine and denatonium but not cycloheximide possessed substantially attenuated activity in later windows (2 to 3 s). The net effect of these response features, as observable from the plots of sequential across-neuron response patterns in Figure 6A, was a difference in early but similarity among late windows of taste activity to cycloheximide and quinine/denatonium across all C3 cells.

This trend of divergent early and convergent later sequences of activity by C3 cells to cycloheximide and quinine/denatonium was captured over multiple stimulus concentrations by dimensionality reduction. The three-dimensional space in Figure 6B plots the outcome of PC analysis of responses by all C3 neurons to each concentration of cycloheximide, quinine, denatonium, SOA, and also water, measured during sequential 500 ms periods of stimulus delivery. Lines connected PC-mapped points for consecutive response windows of the first trial for each concentration of a tastant, which gave a “trajectory” [28], [38], [40] for the time-evolved, across-neuron response to each input. Dissimilar trajectories between stimuli reflected different spatiotemporal activity patterns during taste presentation, whereas similar trajectories reflected correlation in space/time characteristics of responses. Under this approach, time-dependent differences in neural coding were evident for select bitters. In C3 neurons, salient concentrations of cycloheximide evoked similar overall trajectories that initially diverged from those induced by quinine and denatonium (Figure 6B). Strong divergence of these codes was apparent during the epoch 1 to 1.5 s following stimulus onset. Early periods of the trajectories to increasing concentrations of quinine and denatonium systematically followed a similar course in PC space, albeit early periods of paths for these stimuli were clearly shifted away from activity to cycloheximide. Yet later periods of trajectories for responses to cycloheximide, quinine, and denatonium converged onto a common general location in space. Additional PC analysis applied to consecutive 500 ms time windows of activity to cycloheximide, quinine, and denatonium averaged across concentration highlighted these effects (Figure 6C). In C3 neurons, gustatory activity to bitter stimuli differentially evolved: during a 5 s taste presentation, across-neuron activity to cycloheximide and quinine/denatonium initially diverged, but gained similarity during later phases of the evoked response.

**Figure 7:**
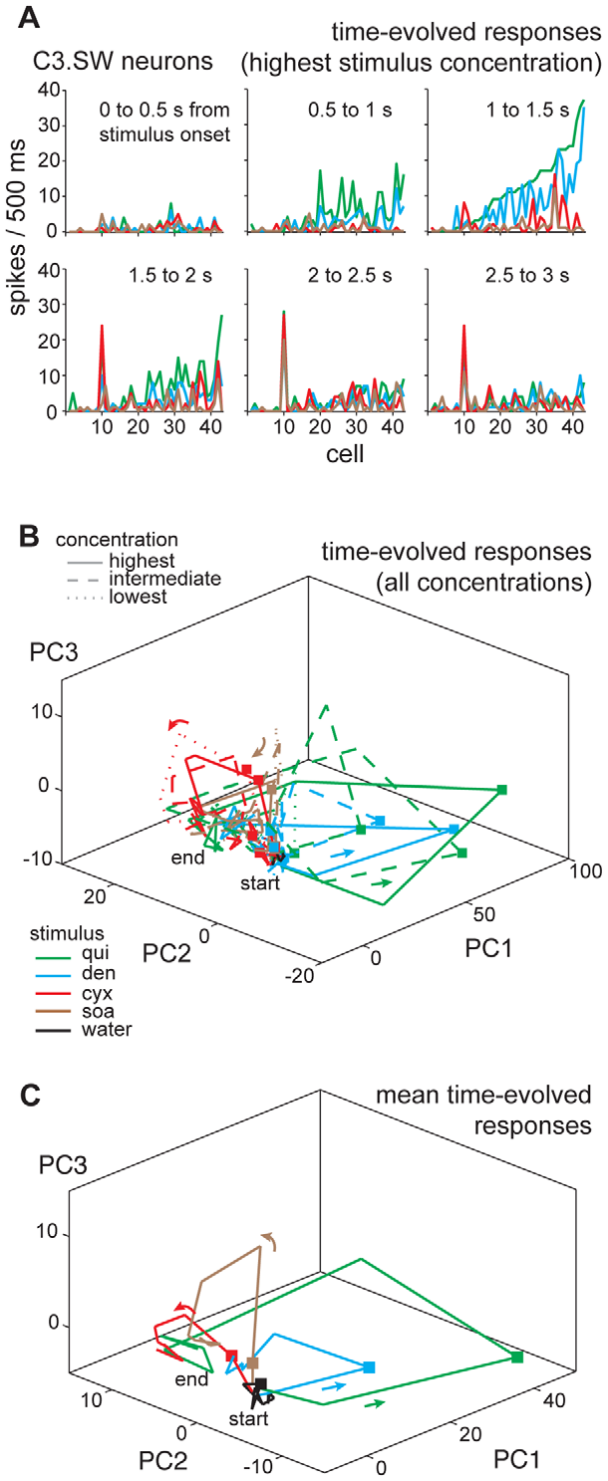
Modeling time dependencies in bitter coding by C3.SW neurons. (**A**) Plots showing sequential, 500 ms wide windows of taste activity (spike density per half-second, ordinates) across 43 C3.SW cells (abscissae) to the highest concentrations of quinine, denatonium, cycloheximide, and sucrose octaacetate. The time window of taste activity captured by each plot is indicated. Legend in **B** gives the stimulus associated with each colored response for all panels in this figure. (**B**) Three-dimensional plot showing the outcome of principal components (PC) analysis applied to sequential, 500 ms wide windows of activity across 43 C3.SW neurons during taste stimulation with all concentrations of quinine, denatonium, cycloheximide, sucrose octaacetate, and also water. Response windows from stimulus onset to offset (i.e., 0 to 5 s post stimulus) are represented. For each stimulus, PC-mapped points for sequential response windows are connected using color-coded lines, as in Figure 6. Arrows indicate flow of contiguous points/response windows; squares mark points for response windows residing 1 to 1.5 s post stimulus onset. Response “paths” for activity to all low, intermediate and high concentrations (legend and Table 1) of each stimulus are shown; responses to intermediate concentrations are not differentiated. PC1, PC2, and PC3 explain 76% of the total response variance. The general locales of the “start” and “end” points for the trajectories are indicated. (**C**) Same as panel **B**, except that activity within each 500 ms window was averaged over concentrations for each stimulus prior to PC analysis, highlighting global trends in the data. PC1, PC2, and PC3 explain 83% of the total response variance.

Similar findings were found for C3.SW neurons. Figure 7A shows consecutive half-second windows of across-neuron activity by all C3.SW cells during oral stimulation with the highest concentration of quinine, denatonium, cycloheximide, and SOA. For all plots, neurons are rank-ordered by their response to quinine in the epoch 1 to 1.5 s following stimulus onset. During this period, quinine and denatonium clearly evoked response patterns that differed from activity to cycloheximide and SOA. In subsequent epochs, many neurons that showed robust early activity to quinine and denatonium gave diminished responses to these inputs, and activity to all bitters began to and eventually converged to a generally common pattern in late windows (2 to 3 s) of taste delivery. As for C3 units, the trend of C3.SW cells to show spatiotemporal responses to bitters that diverged during early but converged in later response windows was captured by PC analysis of bitter activity across multiple stimulus concentrations (Figure 7B) and averaged across concentration (Figure 7C). In summary, C3.SW cells showed responses to cycloheximide and SOA that initially differed from activity to quinine and denatonium. However, activity to all of these inputs showed relatively heightened similarity during later phases of taste delivery.

### Comparing spatiotemporal activity between bitter and non-bitter stimuli

These models indicate that gustatory neurons from both C3 and C3.SW mice show differential spatiotemporal patterns of activity to bitter stimuli, and reveal that differences in early stages of taste processing largely underlie this effect. This could reflect discernable features of sensory codes among “bitter” inputs or, alternatively, non-significant variation in activity that is typical among stimuli of one taste category. To begin to explore this, we compared spatiotemporal neural population responses to bitters with time-evolved activity to stimuli of other quality classes. Comparison stimuli included the Na^+^ salts NaCl and NaNO_3_; NaCl and large anion Na^+^ salts stimulate amiloride-sensitive receptor mechanisms mediating sodium taste quality [47]. We also assessed activity to HCl and citric acid, which are transduced by a common acid receptor thought to drive sour taste quality [48].

**Figure 8:**
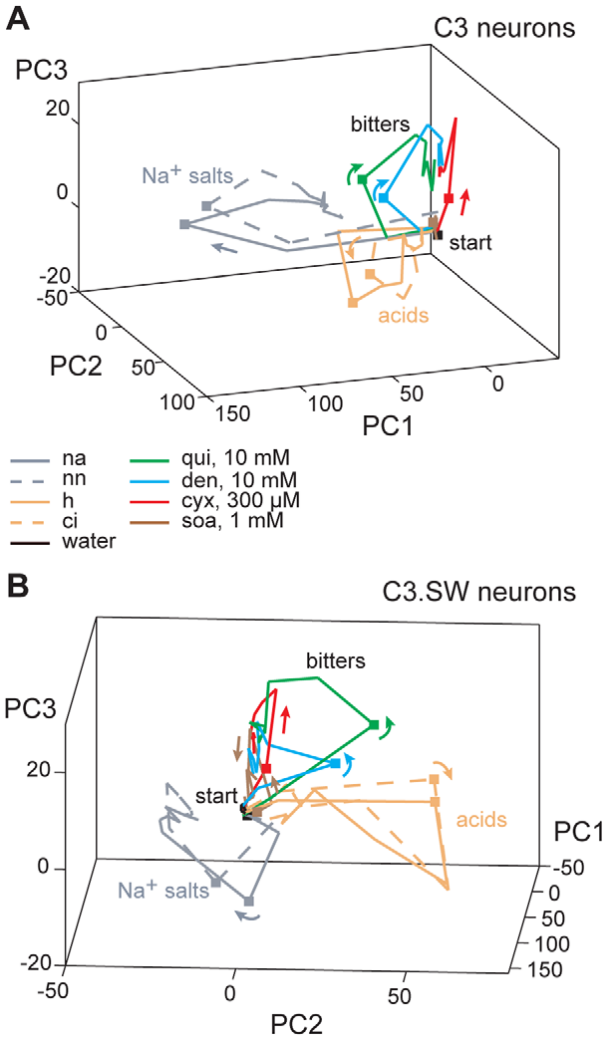
Modeling time dependencies in neural coding for bitter and other stimuli. Three-dimensional plots showing the outcome of principal components (PC) analysis applied to sequential, 500 ms wide windows of activity to bitter tastants, Na^+^ salts, acidic stimuli, and also water across 36 C3 (**A**) and 43 C3.SW (**B**) neurons. Half-second wide response windows from stimulus onset to offset (i.e., 0 to 5 s post stimulus) are represented. For each stimulus, PC-mapped points for sequential response epochs are connected using color-coded lines (legend, Table 1 gives abbreviations), forming “paths” in the space describing time-evolved neural activity to taste inputs. “Elbows” along each path represent points for response windows. Arrowheads indicate flow and sequencing of contiguous windows. The general locale of the “start” for each trajectory in PC space is indicated. Along each path, the point representing time-windowed activity arising 1 to 1.5 s post stimulus onset is marked by a square. Legend in **A** applies to both panels.

**Figure 9:**
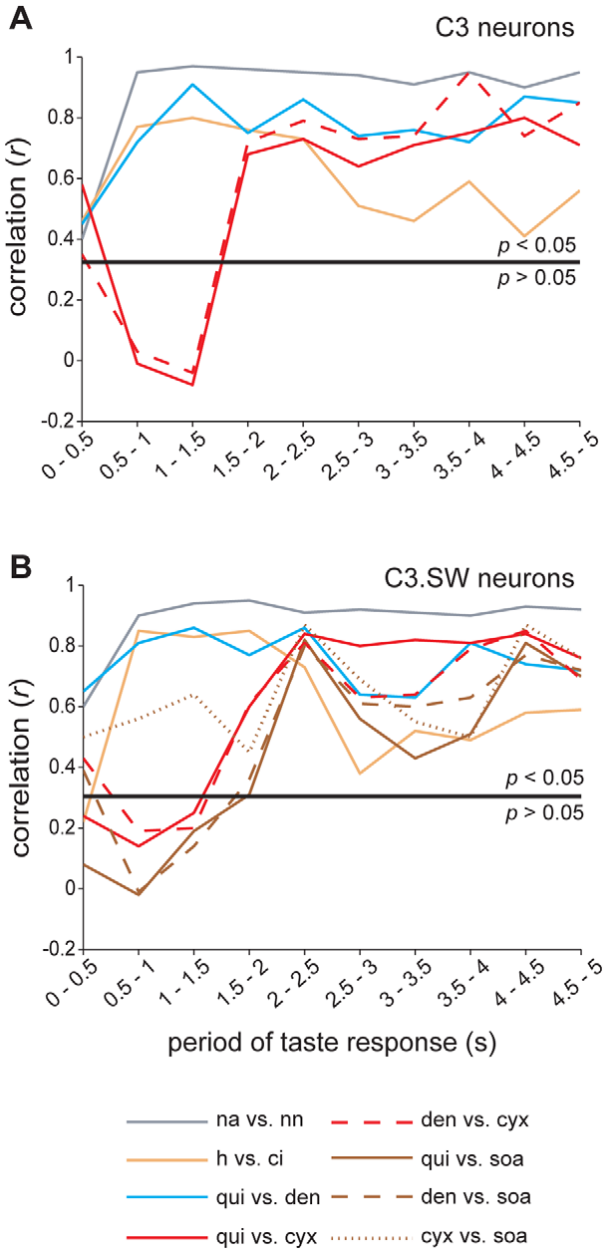
Similarity and dissimilarity among bitter responses through time. Correlations (Pearson's *r*, ordinates) among responses to Na^+^ salts, acids, and bitter stimuli measured during sequential, half-second wide periods of taste responses (abscissae) for 36 C3 (**A**) and 43 C3.SW (**B**) neurons. Legend in **B** gives the stimulus comparison denoted by each trace and applies to both panels. For bitter stimuli, activity to only the highest concentrations (Table 1) is represented. Analyses/plots involving sucrose octaacetate are not shown for neurons recorded from C3 mice, which are insensitive to this stimulus. Solid black line gives the significance criterion for *r* as based on the number of cells.

Na^+^ salts and acids induced similar, within-quality spatiotemporal response patterns in both C3 and C3.SW cells. This was shown by correspondence between PC-mapped response trajectories for these stimuli over half-second wide windows of taste delivery (Figure 8) and confirmed by correlation (*p*<0.05) among responses to Na^+^ salts or acids across contiguous windows of gustatory activity (Figure 9). This sustained intra-quality correlation during taste responding was not found across bitter stimuli. No correlation (*p*>0.05) was observed during early periods of responses to quinine or denatonium compared against cycloheximide and SOA (Figure 9), an effect that followed the early divergence of activity to these stimuli captured by trajectory analyses (Figures 6, 7, and 8). In C3 and C3.SW mice, select Na^+^ or acidic stimuli induced similar spatiotemporal patterns of response across populations of taste-sensitive neurons. On the other hand, different “bitter” stimuli induced different spatiotemporal responses, questioning the singularity of the neural code for bitter inputs.

## Discussion

This study found differences among central neural codes for individual bitter stimuli in two lines of mice and discovered that the early phase of the taste response carries these differences. Our findings are supported by several other studies indicating heterogeneity among neural responses to bitter tastants [15]–[19], [25]. Importantly, our results were obtained using mice with controlled genetic backgrounds. Genetic variation is a consideration for studying the neural representation of bitter tastes, as sensitivities to bitter stimuli can independently vary across heterogeneous subjects. For example, humans can show wide variation in their relative sensitivities to diverse bitter stimuli, with sensitivity to one bitter chemical sometimes not predicting sensitivity to others [20]–[22]. Similarly, genetically heterogeneous outbred rats vary in their relative sensitivities to quinine/denatonium and cycloheximide [23]. Inter-individual differences in sensitivity to bitter stimuli likely reflect allelic variation of independent mechanisms involved in bitter taste detection [20]. The present work using genetically controlled mice aimed to remove inter-individual variation as a factor in the analysis of gustatory neural coding, leaving circuit properties to guide resulting bitter response effects. Our results strongly suggest that divergent codes for bitter stimuli arise in part from an inherent organizational feature of the gustatory pathway.

A general result of our study was that cycloheximide and SOA induced patterns of activity across neurons that were distinct from those to quinine and denatonium. This effect was consistent over several concentrations of these stimuli delivered “whole mouth”. The present data differ from our prior results that revealed strong associations among taste codes for bitter stimuli in rat NTS [13]. However, this previous study pertained to bitter input arriving primarily from the anterior tongue and palate and, unlike the present work, did not attempt to include stimulation of posterior tongue receptive fields.

The present results agree with evidence for differential rostrocaudal oral expression of receptors for quinine/denatonium and cycloheximide/SOA. Gustatory sensation is supplied in part by cranial nerves VII and IX, which respectively innervate TBCs on anterior and posterior oral fields. In mice, oral delivery of quinine evokes robust responses in nerves VII and IX [49], [50] and denatonium can induce responses of like magnitude in both nerves [51]. Similarly in rats, quinine and denatonium are effective for VII and IX [16] and downstream bitter-sensitive NTS neurons supplied by these nerves [13], [19]. On the other hand, oral delivery of cycloheximide or SOA induces strong activity in rodent IX but a relatively low or null response in VII [13], [17], [19], [49]–[52]. Thus, unlike quinine and denatonium, SOA and cycloheximide tend to show high, but possibly not exclusive [53], affinity for receptors innervated by IX. Speculatively, TBCs supplied by VII and IX may express different repertoires of bitter taste receptors. Although such an arrangement could contribute in part to differential neural responses to bitters, more work would be needed to precisely determine how the present findings relate to patterns of expression of taste receptors for bitter stimuli.

Most bitter-responsive neurons we recorded from bitter-sensitive C3 and C3.SW mice showed variable and broad tuning across taste qualities (Figures 2, 3, and 4). Only a few cells in each line demonstrated somewhat selective tuning to bitter stimuli or gave their highest net spiking response to a bitter input. A relative paucity of such “bitter best” cells was also reported in a study of bitter coding in rat NTS where a similar attempt was made to stimulate the whole mouth with stimuli [19]. The tuning properties of bitter-responsive cells found presently raise the possibility that brain stem codes about “bitters” in our mice could be contributed by neurons that display heterogeneous, mostly non-selective gustatory tuning. Similarly in rats, differential activation of diverse gustatory neuron types in the pontine nucleus has been postulated to contribute distinctions among bitter tastants [18]. Furthermore, neurotomy of nerve VII in rats can impair oral sensory discriminations involving bitter stimuli [54] and bitter-sensitive rat NTS neurons supplied by VII are non-selective across taste qualities, showing strong and indiscriminate activity to bitters, salts, and acids [13]. However, it is important to acknowledge that the aforementioned work and present study measured passive features of neural responses to oral stimuli presented under anesthesia. Although promoting measurement of neural activity across animals in the absence of behavioral differences [35], the anesthetized preparation precludes the influence of active sampling (i.e., mouth movements) on neural selectivity. Additional recording studies using awake, behaving animals are needed to continue to explore the tuning of brain stem neurons sensitive to bitter, and other, taste inputs.

Our analyses of time-evolved ensemble responses found that NTS activity to quinine and denatonium was distinct from that to cycloheximide and SOA during only an early window of taste presentation, from approximately 0.5 to 1.5 s post stimulus. NTS responses among bitters subsequently converged to a generally common pattern in later response periods, beginning approximately 2 to 3 s post stimulus. It is important to note that the zero point used to measure response time course (i.e., 0 s) for all neurons was aligned with the computer signal that switched on taste stimulus flow. This mark preceded stimulus contact with oral epithelia and subsequent gustatory activity by close to 400 ms. Thus, there was a brief lag from the zero point to the taste response contributed by this delay, and any additional delay due to stimulus/receptor kinetics. From this, the response alignment and time-windowing methods used overestimated when differences and similarities in neural responses occurred, with effects arising several hundred milliseconds earlier in gustatory-induced activity than captured by our approach. Further, although all mice were stimulated in the same manner to mitigate temporal irregularities in stimulus presentation and response, we cannot rule out the possibility of millisecond-timescale variance in taste response alignment across neurons sampled from multiple mice. Nonetheless, the early divergence among responses to bitter inputs was robust and existed over a broad time period (at least 1 s). Subtle irregularities in trial alignment would likely not impact the capture of this effect.

Differences during early periods of spatiotemporal codes, as observed for bitter stimuli, were not found for pairs of same-quality, non-bitter inputs, such as the Na^+^ salts NaCl and NaNO_3_ and the acidic stimuli HCl and citric acid. Although these data could begin to suggest that intra-quality diversity of neural codes is unique to the bitter category, other possibilities exist. To delineate this concept would require analyses of response time course to several concentration-varied, non-bitter stimuli of each taste category. Such an experiment could also be important for understanding how space and time aspects of central taste processing fit with the classic notion that taste experiences fall into only four or five categories, and whether intra-quality diversity in neuronal responding is more prevalent than usually considered. Along this line, there is evidence from rats that intra-quality taste discrimination, as observed for select bitters [25], can occur between the “sweet” stimuli sucrose and maltose [55], albeit little is known about potential differences in central codes for these sugars.

In rodent cortex, different attributes of taste stimuli are carried by distinct, contiguous phases of taste responses, where a single response can multiplex information through these phases. Specifically, cortical gustatory neurons signal information about a tastant's identity near the beginning of a response (i.e., from approximately 0.2 to 1 s) and later epochs of the same response register a tastant's palatability [56]–[58]. Given the contribution of the beginning phase of taste activity to chemosensory identity, it is tempting to speculate that the early differences among bitter responses found presently in the NTS reflect coding of differences in specificity among “bitter” stimuli. Moreover, the subsequent convergence of NTS responses to bitter tastants in later epochs of taste trials agrees with their common aversiveness and that information about palatability arises in late response windows. However, there are some important considerations to applying this model of multiplexing to the present data. Foremost, cortical data showing sequential coding of specificity-then-palatability were obtained from awake rats that behaviorally responded to tastants, whereas the present study measured NTS activity in anesthetized mice. Comparing neural data across species, brain regions, and behavioral states can be done only with caution, as these variables likely influence neural codes. Further, the type of stimulus information carried across sequential phases of bitter activity is not discernable from the present data. It is plausible that early distinctions among responses to bitter stimuli could reflect a difference between these chemicals unrelated to a qualitative effect, and that convergence of neural codes in later phases of activity signals a common “bitterness”. It is also unknown if the degree of early neural difference in bitter responding captured by our methods would be sufficient to support detection of differences among bitter tastes. To indeed delineate how the present differences in gustatory representations for bitter stimuli relate to potential sensory differences among these inputs requires further studies that involve behavioral discrimination tests in mice, ideally coupled with awake and behaving neural recordings. Nevertheless, rats can discriminate among the tastes of the bitter stimuli nicotine and quinine and show awake cortical responses that associate with this behavior [25], which argues that the taste system does in fact have the means to signal a type of perceptual difference between select “bitter” taste chemicals.

In addition to their ability to discern quinine from nicotine, rats performing in taste detection assays have shown capacity for discriminating quinine from other bitter-like stimuli, such as KCl [54]. On the other hand, rats failed to discriminate denatonium from quinine in taste detection tests [11], which agrees with the high correlation among responses to these stimuli found presently and in other neural studies [13]. Further data on behavioral discrimination among bitter stimuli are limited. Most studies on the perceptions induced by various bitters have not explicitly tested for discrimination: the degree to which perceptual differences between stimuli could be reported. Nevertheless, the outcomes of these studies do suggest there are mechanisms that could support the detection of differences between bitters. For instance, learned aversion generalization studies, which index perceived commonality among stimuli [59], have shown hamsters do not cross-generalize taste aversions between select bitters, such as quinine and SOA [24]. This lack of cross-generalization indicates that these stimuli do not share the same percept and likely engage non-overlapping gustatory processes, as also suggested by the present data. Habituation generalization studies in *M. sexta* showed bitters that activate distinct receptor cells or signaling pathways do not cross-habituate [60], suggesting these stimuli are discriminable to caterpillars. Further, psychophysical studies in mice (e.g. [29], [61]), rats [23], [53], and humans (e.g. [20], [22]) have revealed differences in covariation among sensitivity to bitters under various conditions. Although these experiments did not explicitly address issues of bitter discrimination [20], they do suggest that diverse bitters can be detected by independent mechanisms – a prerequisite for a neural system that could compute perceptual differences among bitter inputs.

Why would the sense of taste “want to” discriminate among “bitter” stimuli? The answer here likely reflects an animal's natural ecological niche [17]. Omnivores and herbivores face a wide range of bitter toxins, including alkaloids and tannins, in their diet of plant foods [62]–[66]. Further, certain bitter toxins possess medicinal properties and animals may seek and consume materials containing these chemicals to reduce maladies [65], [67]. The taste of “bitter” chemicals may associate with nutritive and medicinal value of plants to select animals. It follows that the assumption that “bitterness” presages danger [5] and substances inducing this percept should always be rejected could be a maladaptive behavior for plant-eating animals in the wild [63]. Nonetheless, the toxicity of bitter chemicals found in plants can widely vary [63], [65], [68] and the ability to distinguish among the “tastes” of different bitters would potentially afford discrimination of plant lethality. Such discrimination would support selection of plants of reduced toxicity or signal highly toxic vegetation requiring countermeasures for edibility, such as the co-ingestion of earth performed by certain plant-eating animals to presumably absorb and neutralize bitter toxins in plants [62], [69]. Neural distinctions among the tastes of “bitter” stimuli, as observed presently, may reflect the conservation of a trait supporting perceptual distinctions used in the wild to discriminate chemical toxicity [53]. Although denatonium and SOA are synthetic chemicals that from an evolutionary perspective co-opt bitter taste receptors, the naturally occurring bitters quinine, an alkaloid in vegetation, and cycloheximide, produced by bacteria in soil, were found here to induce differential taste codes in mice, corresponding to a roughly tenfold difference in the toxicities of these chemicals (mouse oral LD_50_: cycloheximide, 0.1 g/kg [70]; quinine, 1.2 g/kg [71]). Given the similarity in response between cycloheximide and SOA, it is furthermore of interest that the closest behavioral “match” for SOA phenotype variation in C3 and C3.SW mice is the extremely toxic alkaloid strychnine [29]. Although some studies show the toxicity of bitter chemicals does not co-vary with their detection thresholds [63], others have revealed ecologically-relevant distinctions among taste codes for natural bitter compounds that do differ in toxicity [72].

The present results agree with data indicating heterogeneity among neural codes for bitter taste stimuli and raise the testable possibility that sensory features of select “bitters” may be discernable by mice, as in other animals. It remains to be determined whether the potentially unique features among bitters, as reflected by their neural codes, might be qualitative or another type of sensory attribute. The developing literature on bitter taste coding warrants further studies on perceptual similarities and differences among “bitter” stimuli. Such work will be important for understanding the relevance of assigning “bitter” stimuli in our taste world, and that of other species, to a unitary taste class.
